# Simple Wastewater Preparation Protocol Applied to Monitor the Emergence of the Omicron 21L/BA.2 Variant by Genome Sequencing

**DOI:** 10.3390/v15020268

**Published:** 2023-01-17

**Authors:** Nathalie Wurtz, Maelle Boussier, Louis Souville, Gwilherm Penant, Alexandre Lacoste, Philippe Colson, Bernard La Scola, Sarah Aherfi

**Affiliations:** 1 MEPHI, Institut de Recherche pour le Développement (IRD), Aix-Marseille Université, 13005 Marseille, France; 2 Institut Hospitalo-Universitaire Méditerranée Infection, 19-21 Boulevard Jean Moulin, 13005 Marseille, France; 3 Assistance Publique—Hôpitaux de Marseille (AP-HM), Aix-Marseille Université, 13005 Marseille, France; 4 Bataillon des Marins Pompiers de la ville de Marseille, 13005 Marseille, France

**Keywords:** SARS-CoV-2, variant, Omicron, sewage, protocol, qPCR, next-generation sequencing, genomics

## Abstract

Detecting and monitoring viruses in wastewater samples have been reported as useful ways of tracking SARS-CoV-2 epidemic trends. However, there is currently no unanimously recognised method of processing samples to identify and quantify SARS-CoV-2 variants in wastewater. We aimed to implement a method that was as simple as possible in order to be used universally. In a study performed between January 2022 and June 2022 in the city of Marseille, France, we first evaluated the impact of the sample preservation strategy. We then compared ultracentrifugation to ultrafiltration and several steps of filtration to determine the optimal approach for virus concentration. As a proof-of-concept, the definitive protocol was applied to next-generation sequencing of SARS-CoV-2 in wastewater to monitor the emergence of the Omicron variant in the city. For sewage water to be processed in the week following the sampling, storage at +4 °C is sufficient, with less than 1 Ct loss. Filtration with a 5 µm syringe filter, then with a 0.8 µm filtration unit, followed by ultrafiltration was the optimal protocol, leading to an average increase of 3.24 Ct when the starting Ct was on average 38 in the wastewater. This made it possible to observe the emergence of the Omicron 21L/BA.2 variant after Omicron 21K/BA.1 by genome sequencing over a period ranging from 20 February to 10 April 2022 in agreement with observations based on patient data. To conclude, by using a simple method requiring only basic filters and a centrifuge as equipment, it is possible to accurately track the relative incidence rates and the emergence of SARS-CoV-2 variants based on sewage samples.

## 1. Introduction

The emergence of new SARS-CoV-2 variants, associated with either increased transmissibility, escaping antibody responses, or both, have recently attracted attention [1,2]. As of September 2022, several variants have now spread around the world [3,4,5]. To date, one of the most recent of these is the Omicron variant (B.1.1.529), which arrived from South Africa [6] including sub-lineage variants such as 21L (Nextstrain classification (https://clades.nextstrain.org/; access date: 16 January 2023)) or BA.2 (Pangolin classification (https://cov-lineages.org/resources/pangolin.html; access date: 16 January 2023)), which was first highly prevalent in Denmark, then spread to across the whole of Europe [7,8]. Monitoring these variants within a community is necessary to understand their transmission dynamics and take appropriate public health measures. This surveillance is currently mainly performed using real-time reverse transcription (RT)-PCR (qPCR) or whole genome sequencing from nasopharyngeal samples from infected patients. This approach is relatively expensive, labour intensive, and time consuming, and cannot be exhaustively implemented in all settings such as low resource environments. In addition, this approach presents a major bias, since only the genomes of symptomatic people who have attended hospital or presented at a laboratory to be tested for SARS-CoV-2 by RT-PCR and who presented well-defined criteria were sequenced and analysed. This approach thus overlooks asymptomatic infections that may represent more than one-third of SARS-CoV-2 infections, as suggested by one recent study [9].

Several studies have reported the presence of SARS-CoV-2 RNA in stool and anal/rectal swabs, not only from symptomatic but also from asymptomatic patients [10,11,12]. It should be noted that even if the faecal–oral transmission of SARS-CoV2 has not been clearly demonstrated, viruses in stools can rarely still remain infectious [13,14]. Therefore, the analysis of SARS-CoV-2 in wastewater would appear to be an efficient approach to monitoring the burden of disease and infection in the community. Monitoring the SARS-CoV-2 epidemic through wastewater may also make it possible to more easily monitor particular establishments such as nursing homes and hospitals, particular districts in a city, or may even make it possible to assess the effects of sanitary measures and lockdowns that may be imposed in the context of the COVID-19 pandemic [15,16].

The detection and monitoring of SARS-CoV-2 in wastewater samples has been reported in many countries [15,17,18,19,20,21]. Nevertheless, few studies have performed SARS-CoV-2 genome sequencing on sewage samples in order to determine the strains circulating within a community and to study their genetic diversity [16,22,23,24,25,26,27,28,29]. Unexpectedly, some sequences were not identified in clinical samples [20,22,23,24,25,26,30,31]. This method could, therefore, also be a more efficient, faster, and more realistic method of monitoring the emergence and spread of variants within a community. 

Wastewater surveillance of SARS-CoV-2 comprises several phases, starting with the sampling of wastewater and possibly storing wastewater before processing, followed by virus concentration, RNA extraction, SARS-CoV-2 RNA detection, and quantification (qPCR) as well as the potential sequencing of any variants present. Although most studies agree that collected wastewater must be stored at low temperatures (usually at 4 °C) to preserve the viral load [27,28], a large panel of procedures is available that has been proposed to concentrate viruses. Numerous studies have evaluated procedures concentrating on enveloped viruses from wastewater including ultracentrifugation [24,32], ultrafiltration [32,33,34,35,36,37], polyethylene glycol (PEG) and salt-based precipitation [32,35,36,38], skimmed milk flocculation [36,37], sludge extraction [36], bag-mediated filtration [36], and magnesium- and aluminium-based adsorption [34,38]. In a previous study, we used the ultracentrifugation method to concentrate SARS-CoV-2 in wastewater [24]. However, this method requires expensive equipment (an ultracentrifuge), which may not be available in a standard laboratory. Moreover, as the laboratory in charge may be far from the sampling place, it is important to ensure good preservation of samples. In this study, we first evaluated the best method for preserving wastewater samples to avoid the progressive loss of viral RNA, then tried to determine the best method of concentrating the viral RNA present in sewers using a technique that could be implemented in any laboratory.

## 2. Materials and Methods

### 2.1. Wastewater Collection

The study was performed between January 2022 and June 2022 in the city of Marseille in south-eastern France. The SERAMM (Marseille Metropole Sanitation Department) collected a daily sample of 250 mL of wastewater from the separate sewer networks (indicated “RS”) using an automatic “ASP-Station 2000 RPS20B” sampler (Endress Hauser, Huningue, France). This network, referred to as the “RS network”, drains wastewater from Marseille and 16 other municipalities including nearly all hospitals in the city as well as COVID-19 dedicated units. Marseille city has more than 870,000 inhabitants. The wastewater treatment plant capacity of Marseille is one of the most important in France and Europe, servicing 1.82 million equivalent inhabitants. This type of sampling made it possible to fill a refrigerated flask with 20 litres of wastewater per 24 h, collected between 8 am and 8 am the following day. Every day, samples were transferred on ice to the nuclear, radiological, biological, chemical laboratory (the “NRBC unit”) of the Bataillon de Marins-Pompiers de Marseille (BMPM), treated within one hour of collection for multiplex qPCR, then transferred directly to the University Hospital Institute Méditerranée Infection for immediate use.

### 2.2. Preservation of Wastewater Samples

A quantity of 10 mL of wastewater was immediately filtered using 5 μm sterile syringe filters (Clearline, Merck, Darmstadt, Germany). Half of the samples were kept at room temperature while the other half were placed immediately at 4 °C. The study was performed on seven wastewater samples corresponding to seven different dates (seven time points). The quantity of SARS-CoV-2 present in the wastewater was measured on the day of sampling then every week for three weeks. The loss of virus quantity compared with the initial quantity was assessed by calculating the differences between the qPCR cycle threshold values (Delta Ct).

### 2.3. RNA Extraction and RT-qPCR

Nucleic acid extraction and qPCR were performed as previously described [24]. Briefly, the extraction of viral nucleic acids was performed using the EZ1 Virus Mini Kit (Qiagen, Hilden, Germany), following the manufacturer’s recommendations using 200 μL of wastewater and eluted in 60 μL of elution buffer. Real-time RT-PCRs were carried out specifically targeting the N-gene, using the primers previously described [24]. A dilution series of the calibration range of the PCR, tested in triplicate, provided an efficiency of 1.99, a R^2^ of 0.9987, and a SD between the replicates <0.167 for each point. The RT-PCR was carried out using the Superscript III Platinum One-step Quantitative RT-PCR system with the ROX Kit (Invitrogen, Carlsbad, CA, USA) following the manufacturer’s recommendations, with a final concentration of 400 nM of primers, of 200 nM of probe, in a final volume of 20 μL with 5 μL of RNA. The RT-PCR program was performed according to the manufacturer’s recommendations, and carried out on a LightCycler 480i (Roche Diagnostics, Meylan, France). 

An external control based on a SARS-CoV2 strain and calibrated at 30 Ct, with a tolerance of +/− 0.5Ct for this N-gene targeting system, allowed us to control the standardization of all the PCR runs. A negative template control was also put in each PCR run. 

### 2.4. Wastewater Filtration and Concentration Method

#### 2.4.1. Comparison of Ultracentrifugation and Ultrafiltration

The first steps were comparable to that set out in our previous study [24]. Briefly, if large particles were present in wastewater, a low-speed centrifugation step was conducted at 4000× *g* for 10 min at 4 °C. Then, 150 mL of wastewater was serially filtered through a 5 µm polycarbonate membrane filter (Merck Millipore, Burlington, MA, USA) using a vacuum filtration flask, a 0.8 µm disposable filter unit (Thermo Scientific, Waltham, MA, USA), a 0.45 µM bottle-top vacuum filtration system (VWR, Radnor, PA, USA), and finally, a 0.2 µM sterile syringe filter (ClearLine, Dutscher, Bernolsheim, France). For the next steps in comparing ultracentrifugation to ultrafiltration, 50 mL of the filtered samples was centrifuged at 100,000× *g* for two hours using a Sorvall Discovery 90SE ultra-centrifuge. The pellet was resuspended in 200 µL of PBS. At the same time, 50 mL of filtered samples was introduced into Centricon tubes (Centricon Plus centrifugal filter, 70 Merck Millipore, Burlington, MA, USA), then centrifuged for 30 min at 3000× *g*. The Centricons were turned over and centrifuged for two minutes at 1000× *g*. About 200 μL of the eluate was recovered. These tests were carried out in quadruplicate.

#### 2.4.2. Comparison of Filtration Steps

To optimise the viral concentration of SARS-CoV-2 from wastewater, tests on the usefulness of the different filtration steps, based on a previous study [24], were carried out in order to simplify the filtration procedure. Filtration steps at 5 µm, 0.8 µm, and 0.2 µm were performed on a 33 mm filter syringe, while 0.45 µm was performed on the 500 mL filtration units. Concentrations of SARS-CoV-2 were compared using three different protocols. We first tested the filtration at 5 µm, 0.8 µm, 0.45 µm, and 0.2 µm, plus ultrafiltration versus filtration on 5 µm, 0.8 µm and 0.2 µm, plus ultrafiltration (quadruplicate), then filtration at 5 µm, 0.8 µm, and 0.2 µm, plus ultrafiltration versus filtration at 5 µm and 0.8 µm plus ultrafiltration (15 replicates). In a final assay, we compared the filtration at 5 µm, 0.8 µm, plus ultrafiltration, but replaced the 0.8 µm syringe filter with a 0.8 µm filter unit (12 replicates).

### 2.5. Sequencing of SARS-CoV-2 in Wastewater and Sequence Analysis

#### 2.5.1. RT-PCR

Reverse transcription was performed using the SuperScript VILO cDNA Synthesis Kit (11754-250, Thermo Fisher, Waltham, MA, USA), with 10 µL of RNA from each wastewater concentrated sample according to the manufacturer’s protocols. The programme was 25 °C for 10 min, 42 °C for 120 min, and 85 °C for five minutes. The provided cDNA was amplified with the two primer pools (ARTIC nCoV-2019 V3 Panel and 500rxn of IDT 10006788, Integrated DNA Technologies, Inc., Coralville, IA, USA) in 16 wells (8 wells for each pool). The PCR reaction mixture was prepared with 2.5 µL of cDNA template, primer pool (1.6-µM final concentration), dNTP (0.2-mM final concentration), 10 µL of 10× PCR Buffer containing 15-mM MgCl2 (1X final concentration), HotStarTaq DNA Polymerase (Qiagen 203205, Hilden, Germany) (2.5 U per reaction), and DEPC-treated water (qsp 25 µL). The amplification programme was 95 °C for 15 min, followed by 50 cycles, each cycle consisting of denaturation at 95 °C for 30 seconds, annealing at 65 °C for 30 seconds, and extension at 72 °C for 5 min. The program included a final extension step at 72 °C for 10 min. The PCR products (total volume = 400 µL) were concentrated by Nucleofast 96P (Macherey Nagel ref 743100.50, Hoerdt, France) following the manufacturer’s recommendations for a total elution volume of 30 µL for each pool and were separated using electrophoresis in 2% agar gel and visualised with SYBR safe (Invitrogen S3102, Waltham, MA, USA). The Monarch^®^ DNA Gel Extraction Kit (New England BioLabs, Ipswich, MA, USA; ref T1020L, Evry-Courcouronnes, France) was used for the purification of an interest band at 400 pb with an elution at 40 µL for each pool.

#### 2.5.2. Sequencing

The libraries were prepared using the Illumina COVIDSeq protocol (Illumina Inc., San Diego, CA, USA). Each previously purified PCR product was processed for tagmentation and adapter ligation using IDT for the Illumina Nextera UD Indices. Enrichment and clean-up were performed as per the protocols provided by the manufacturer (Illumina Inc., San Diego, CA, USA). All libraries were pooled together, and the pooled samples were quantified using a Qubit 2.0 fluorometer (Invitrogen Inc., MA, USA). Fragment sizes were analysed in an Agilent Fragment analyser 5200 (Agilent Inc., Santa Clara, CA, USA). Sequencing was performed on the Illumina MiSeq instrument (Illumina Inc., San Diego, CA, USA). Negative template controls were performed for each sequencing run.

#### 2.5.3. Bioinformatics

The reads generated from the wastewater samples were analysed as previously described [24]. Briefly, reads from pool1 and pool2 provided by the ARTIC procedure were mapped together against the Wuhan-Hu-1 SARS-CoV-2 isolate genome (GenBank accession number NC_045512.2) using the CLC genomics software v7.5 (Qiagen Digital Insights, Hilden, Germany) with the default parameters. To consider a position, a minimum coverage of 10 reads was considered. The non-synonymous mutations present in more than 10% of the reads were considered. A custom Python script was used to associate mutations present in the output file generated by the CLC genomics workbench with a database of mutations of SARS-CoV-2 variants built by our institute based on the Nextstrain tool [39]. The script generated the mutational events detected, their frequency, the numbers of reads covering a given nucleotide position, the frequency of mutational events for each variant, and the list of variants associated with the given mutation. Then, these results were used to generate graphics with the plotly packages [40,41].

Proportions of reads harbouring mutations specific to the Omicron 21K/BA.1 or BA.1.1 lineage on one hand, and mutations specific to 21L/BA.2 on the other hand, were more deeply observed.

#### 2.5.4. Clinical Samples

Data obtained here from the sewage samples were compared to those obtained from the patients’ respiratory samples and produced in the framework of the EMERGEN consortium led by the French Ministry of Solidarity and Health and Santé Publique France (https://www.santepubliquefrance.fr/dossiers/coronavirus-covid-19/consortium-emergen; access date 16 January 2023). Briefly, these data were generated from SARS-CoV-2-diagnosed patients through screening using the TaqPath COVID-19 Kit (Thermo Fisher Scientific, Waltham, MA, USA), which targets viral genes ORF1, N (nucleocapsid), and S (spike), and using qPCR assays specific to variants that target spike mutations L452R and K417N. Negativity for L452R, and positivity for K417N with positive signals by the TaqPath COVID-19 Kit for all three genes targeted indicated a 21L/BA.2 variant; conversely, a 21K/BA.1 was indicated by the negativity of S gene detection by the TaqPath COVID-19 Kit. When performed, definitive identification of the variant was reached through analysis of the viral genome obtained by next-generation sequencing with the Illumina COVID-Seq protocol on the NovaSeq 6000 instrument (Illumina Inc.), followed by sequence read processing and genome analysis, as previously described [42].

### 2.6. Statistical Tests

In order to compare the two groups of samples with regard to the conservation of wastewater samples (4 °C and room temperature), a paired non-parametric Student T test was carried out (Wilcoxon matched pairs signed rank test). In order to compare the effects of the filtration steps, paired parametric Student T tests were carried out. A verification of the normal distribution was carried out before further analyses in order to identify the Gaussian distribution of the samples (using a Shapiro–Wilk normality test or an Agostino and Pearson normality test, depending on the number of samples). Sample groups were considered statistically different if the p-value was less than 0.05. Statistical tests were performed using GraphPad Prism 7.00 software (https://www.graphpad.com/scientific-software/prism/; access date: 16 January 2023).

## 3. Results

### 3.1. Conservation of Wastewater

**Figure 1:**
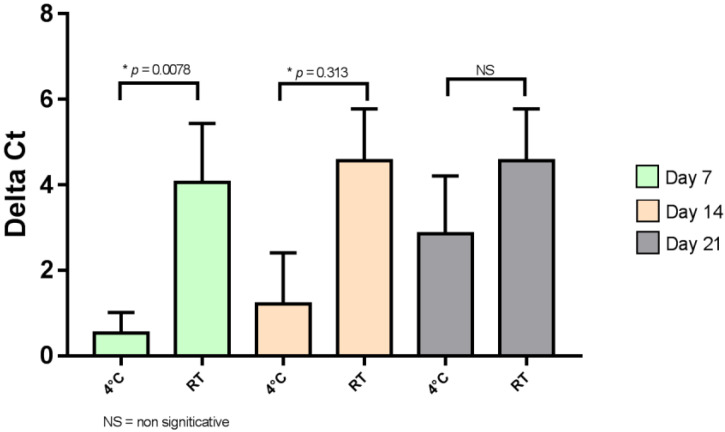
Effect of temperature storage on SARS-CoV-2. NS, not significant; RT, room temperature. * indicate significant differences (*p* < 0.05).

The results of the quantity of virus present in each of the two conditions (4 °C and room temperature) are presented in Figure 1 to compare the effect of storage temperature.

On days 7 and 14, viral RNA was significantly better preserved at 4 °C than at room temperature (*p* < 0.05 at 7 and 14 days of conservation). Moreover, the viral load decreased drastically after 21 days of storage, even when the samples were stored at 4 °C.

### 3.2. Comparison of Concentration Methods

**Figure 2:**
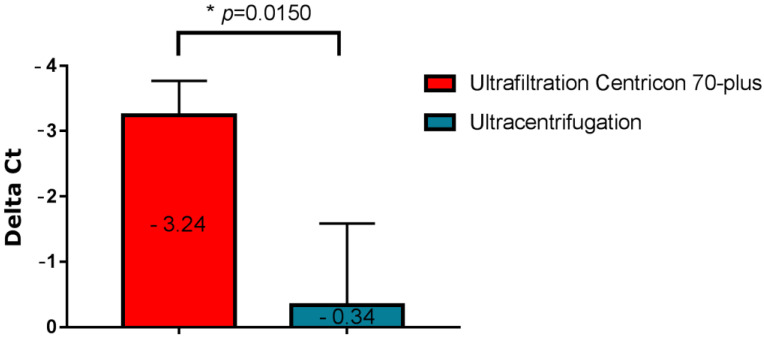
Variation in the amount of SARS-CoV-2 virus in wastewater following two concentration methods; mean Ct ranged from 38 (initial concentration) to 34.8 (ultrafiltration). * indicate significant differences (*p* < 0.05).

Two different concentration methods of SARS-CoV-2 were tested from wastewater: ultracentrifugation and ultrafiltration with a Centricon filter and centrifuge. The comparison of these two concentration methods is shown in Figure 2, which represents the variation in the amounts of SARS-CoV-2 in wastewater after the two concentration methods had been tested.

The method using ultrafiltration with Centricons was more efficient, with an average Delta Ct gain from the native samples of 3.24 compared to the ultracentrifugation method which provided an average Delta Ct of only 0.34. The difference between the two methods was statistically significant (*p* = 0.0150). Thus, concentration by ultrafiltration with Centricons was used for the subsequent tests.

### 3.3. Comparison of Filtration Steps

**Figure 3:**
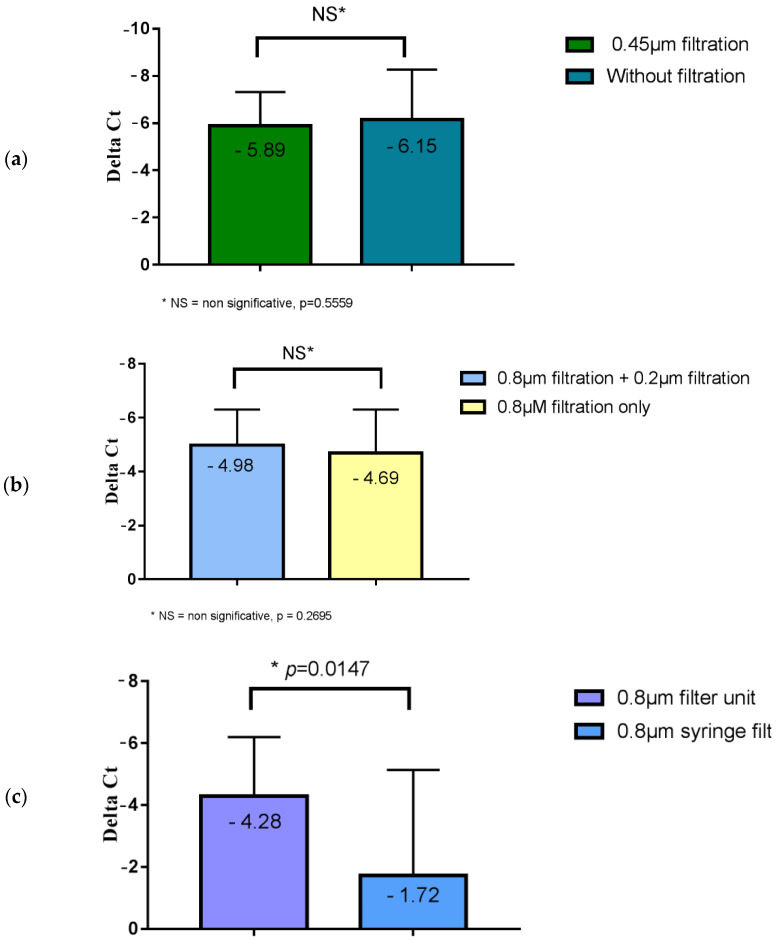
Variation in the SARS-CoV-2 RNA load in wastewater after different filtration steps followed by ultrafiltration: (**a**) comparison of the viral load obtained after filtration on a 0.45 µm filter of wastewater or for unfiltered water followed by ultrafiltration; the mean Ct ranged from 38.1 (initial concentration) to 31.9 (ultrafiltration only); (**b**) comparison of viral load obtained after the filtration of wastewater using a 0.8 µm filter and a 0.2 µm filter, and a 0.8 µm filter only, followed by ultrafiltration, where the mean Ct ranged from 36.2 (initial concentration) to 31.3 (0.8 μm + 0.2 μm filtration + ultrafiltration); (**c**) comparison of the viral load obtained after filtration using a 0.8 µm syringe filter and a 0.8 µm wastewater filtration unit, followed by ultrafiltration, where the mean Ct ranged from 37.6 (initial concentration) to 33.3 (0.8 μm filtration unit + ultrafiltration). * indicate significant differences (*p* < 0.05). NS indicates no significant difference (*p* > 0.05).

We first compared the viral load obtained after filtrations using 5 µm then 0.8 μm filters, followed or not followed by a filtration step on a 0.45 μm filter, and then on a 0.2 µm filter. The results are shown in Figure 3a.

No statistically significant difference between the 0.45 μm filtered and unfiltered wastewater samples was observed (*p* = 0.5559). The filtration step at 0.45 μm was therefore not useful.

We then compared the viral load obtained after filtrations using 5 µm and 0.8 μm filters followed or not followed by a final step with a 0.2 μm filter. The results are shown in Figure 3b. No statistically significant difference was observed between the samples of wastewater filtered on 0.8 μm and 0.2 μm, and those filtered on 0.8 μm only (*p* = 0.3695). The filtration step on 0.2 μm was therefore unnecessary.

Finally, we compared the viral load obtained after filtration on a 5 µm filter, then either on a 0.8 μm syringe filter or on a sterile 0.8 μm filtration unit. The results are shown in Figure 3c. The viral load obtained using filtration units was significantly higher than those obtained with a syringe filter (difference of 3 Ct; *p* = 0.0147).

Hence, the only useful filtration that increased the virus concentration was the filtration step on a sterile 0.8 μm filtration unit.

### 3.4. Sequencing SARS-CoV-2 from Sewers

**Figure 4:**
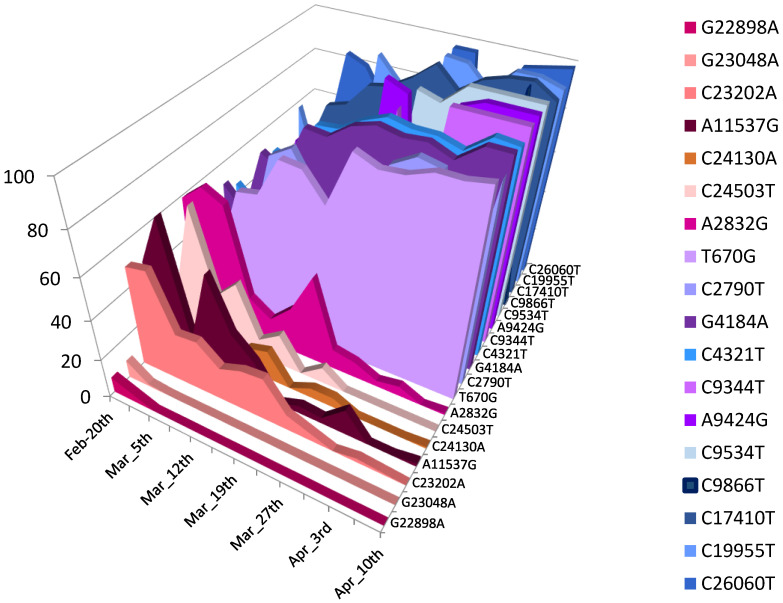
Mean proportions of specific mutations harboured by the Omicron 21K/BA.1 or BA.1.1 lineages and by the Omicron 21L/BA.2 lineage. Mutations A2832G; A11537G; G22898A; G23048A; C23202A; C24130A; and C24503T were found in 21K/BA.1 and in BA.1.1. but absent in 21L/BA.2. Mutations T670G; C2790T; G4184A; C4321T; C9344T; A9424G; C9534T; C9866T; C17410T; C19955T; and C26060T were found in 21L/BA.2 and absent in 21K/BA.1 and BA.1.1.

For each concentrated wastewater sample, a total of 80 μL (40 µL for each pool) of purified DNA extract was sequenced over a three-month period from February 2022 to April 2022. A total of 13 samples was collected on 20 and 22 February, 5, 6, 12, 13, 19, 26 and 27 March, and 2, 3, 9, and 10 April. Mapping on the reference genome of NGS reads provided good SARS-CoV-2 genome coverage, ranging from 87.13% to 99.44% of the genome length. Mean depth of coverage ranged from 28 (for sample collected on 6 March) to 275 (for sample collected on 9 April). The Omicron 21K/BA.1 and 21L/BA.2 variants were clearly predominant throughout the study period. When considering the mutation patterns of 21L/BA.2 that were absent in 21K/BA.1 and BA.1.1, they represented 36–50% of the total number of reads on 20 February, reaching 95–100% on 10 April (Figure 4).

### 3.5. Sequencing SARS-CoV2 in Clinical Samples

**Figure 5:**
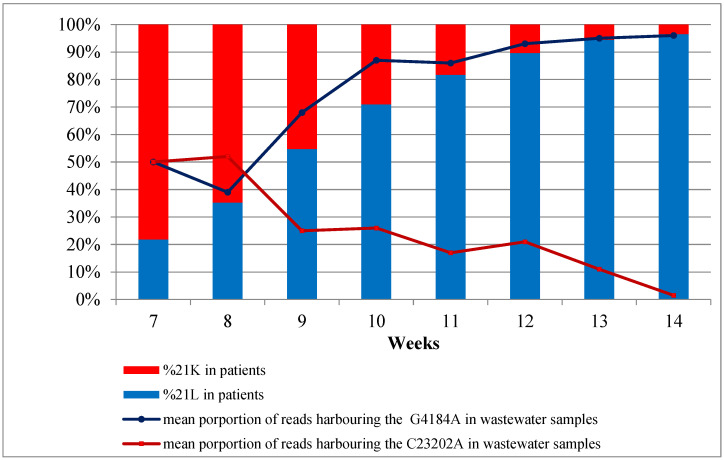
Proportions of reads identified from the 21K/BA.1 (red) and 21L/BA.2 (blue) variants in the patients (histograms) and wastewater (curves) samples. The curves represent the proportion of reads harbouring the C23202A (red) or the G4184A (blue) mutations specific to the 21K/BA.1 and 21L/BA.2 variants, respectively.

We retrieved the SARS-CoV-2 sequencing data from 9,158 clinical samples collected between 14 February and 10 April 2022 and produced in the framework of the EMERGEN consortium (https://www.santepubliquefrance.fr/dossiers/coronavirus-covid-19/consortium-emergen, access date: 16 January 2023). On week 8, 21K/BA.1 and 21L/BA.2 were respectively present in 77.0% and 21.7% of the patients, reaching 3.5% and 96.2% on week 15, respectively. Other genotypes identified included the 21I, 21J, and 21M lineages, but in negligible proportions. These results were correlated with the variant identifications based on SARS-CoV-2 sequencing from the wastewater samples (Figure 5).

## 4. Discussion

Wastewater surveillance of SARS-CoV-2 involves a succession of steps, starting with wastewater collection followed by transport, storage, +/− virus concentration methods, RNA extraction, and SARS-CoV-2 detection/quantification/sequencing. All of these steps can influence the reliability of the results, as previously described [32,43,44]. A major part of the improvements into research on wastewater has focused on extraction and quantification methods [38,45]. However, a better understanding of sample storage and concentration methods will help to improve the detection and sequencing of SARS-CoV-2.

Previous studies have shown that the storage of wastewater samples at 4 °C had no significant effect, while storage at −20 °C or −80 °C led to a significant reduction in the copy numbers of SARS-CoV-2 RNA [46,47]. First, in the present study, we evaluated the effect of the storage of wastewater samples at 4 °C and at room temperature. We showed that wastewater samples should be stored at 4 °C for short-term preservation (<14 days). A refrigerator is therefore the only equipment needed to store wastewater and can be installed in any standard laboratory.

**Figure 6:**
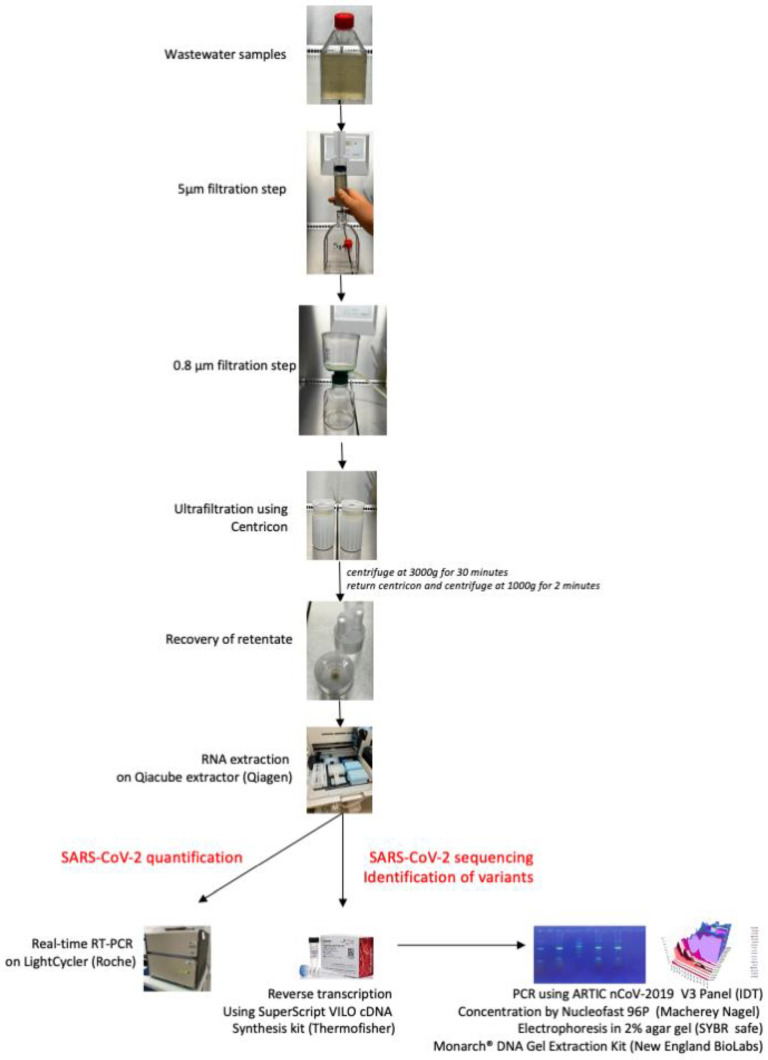
Photographic summary of the definitive protocol for the preparation of wastewater samples.

Second, wastewater samples must be sufficiently concentrated to allow for the effective detection of very few viruses in a large volume of water. Several studies have evaluated the best method of concentrating enveloped viruses in wastewater [26,35,36,38]. For SARS-CoV-2, a preliminary study carried out in our laboratory showed that ultracentrifugation was the best way to concentrate the virus [24]. However, this method requires expensive equipment (an ultracentrifuge) that is available in research laboratories but almost never in laboratories performing routine clinical biology or wastewater analyses. In this study, the ultrafiltration concentration method was able to concentrate SARS-CoV-2 RNA more efficiently than ultracentrifugation. The individual tests for each filtration step showed that filtration at 0.8 μm using a sterile filtration unit after filtration at 5 µm was the only procedure that had a real impact on the viral concentration. This simple procedure for concentrating and filtering wastewater samples can be carried out in any laboratory, since a simple centrifuge is all that is needed. A photographic summary of the different stages is presented in Figure 6.

Results from the whole genome sequencing of the wastewater samples enabled the detection and discrimination of the specific mutations of the two predominant variants, 21K/BA.1 and 21L/BA.2, which were circulating during this period. Analysis of the proportions of the reads harbouring the specific mutations of these two variants in the sequential wastewater samples made it possible to track the quasi extinction of the 21K/BA.1 variant and the rapid emergence of 21L/BA.2 over time. These simultaneous evolutions, with the curves crossing at week 8 of 21K/BA.1 and 21L/BA.2, were superimposable and correlated with those found based on the analysis of respiratory samples from patients. These results should probably be weighted according to the transmissibility of the viral variants. Indeed, the SARS-CoV-2 load in the stool collected from a patient infected by a highly transmissible variant will be higher than that for a patient infected by a less transmissible variant. For the 21K/BA.1 and 21L/BA.2 variants, it has been shown that the mean Ct obtained from 21L/BA.2-infected patients was lower than those obtained from the 21K/BA.2-infected patients [48,49]. It has been previously demonstrated that rainfall may impact the SARS-CoV2 concentration in wastewater [50]. It is noteworthy that the RS network of Marseille collects only domestic wastewater; storm water being collected in a different and adapted network. Moreover, average rainfall amounts in Marseille range from 500 to 700 mm per year. Between 20 February and 10 April 2022, the rainfall amount was low, reaching only 6.4 mm distributed on 3 days (13, 20, and 30 March).

In addition to SARS-CoV-2, the proposed protocol could make it possible to simply process wastewater samples in developing countries or during occasional studies in various areas of the world without the need for very complex equipment. Similarly, this protocol should probably be tested for monitoring other respiratory viruses, gastroenteritis, or even arboviruses when they are excreted in urine and/or stool.

## Data Availability

Not applicable.
